# The molecular determinants of phenotypic plasticity in homeostasis and neoplasia

**DOI:** 10.47248/chp2401020010

**Published:** 2024-12-13

**Authors:** Bradley Balk, David W. Goodrich

**Affiliations:** 1. Department of Pharmacology and Therapeutics, Roswell Park Comprehensive Cancer Center, Buffalo, NY 14263, USA;; 2. Department of Urology, Roswell Park Comprehensive Cancer Center, Buffalo, NY 14263, USA

**Keywords:** cancer, plasticity, heterogeneity, epigenetics, homeostasis, therapeutic resistance

## Abstract

Phenotypic plasticity, the capacity of cells to transition between distinct phenotypic and lineage states over time, is a genetically and epigenetically encoded trait essential for normal development and adult tissue homeostasis. In cancer, phenotypic plasticity programs can be deployed aberrantly to enable disease progression and acquired therapeutic resistance. Cancer phenotypic plasticity is a current barrier to achieving cures for advanced cancers using available molecularly targeted therapies. This review summarizes the complex and interconnected molecular pathways implicated in phenotypic plasticity, both in the context of normal tissue homeostasis and cancer. Molecular pathways convergent between these contexts are highlighted while pathways enabling plasticity are distinguished from those that specify the phenotype of already plastic cells. Key unresolved questions in the field are discussed along with emerging technologies that may be used to help answer them.

## 1. Introduction

**Figure 1.**
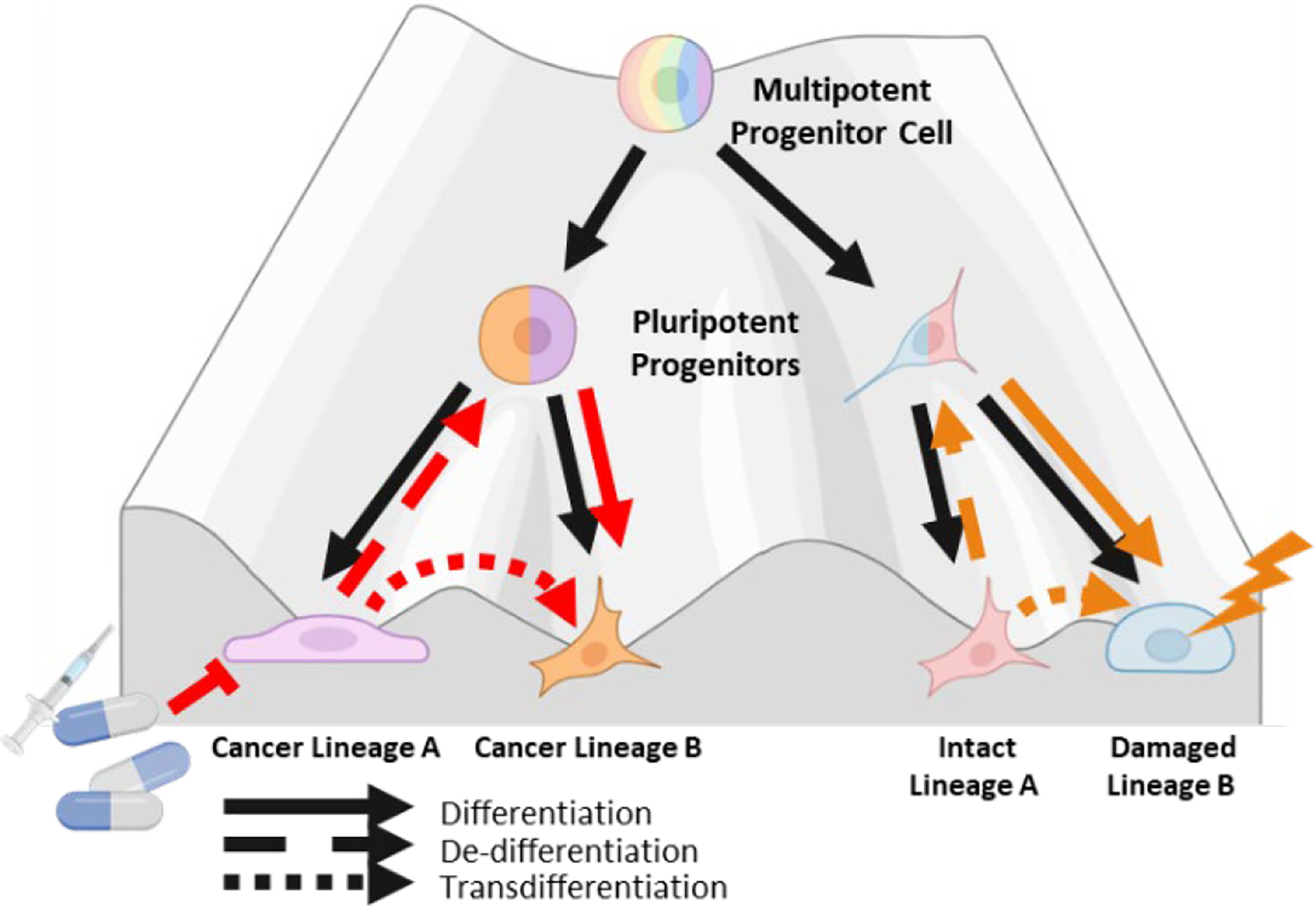
Waddington’s landscape model of cell state commitment. Waddington’s metaphor for cell fate commitment describes cells as marbles rolling down a hill, becoming increasingly differentiated as they reach valleys at the bottom. Valleys represent stable phenotypes. Arrows in black depict cellular differentiation toward a committed cell state while arrows in red depict routes by which committed cells can change cell state due to phenotypic plasticity. This metaphor is applied to normal tissue homeostasis on the right, where damaged cells of lineage B can be regenerated from cells of lineage A. On the left, the metaphor is applied to cancer cells where cancer lineage A is targeted therapeutically, and these cancer cells can convert to a therapeutically resistant cancer lineage B by deploying phenotypic plasticity programs. Created using BioRender.com

Waddington’s landscape model for cell fate commitment during differentiation depicts cells as marbles rolling down a figurative hill of differentiation towards a committed cell state (Figure 1) [1]. This metaphorical hill has many forking paths meant to represent the multitude of different cell fates totipotent stem cells can assume. Waddington’s metaphor implies that cellular differentiation is generally unidirectional and stable once cells assume a committed differentiated state at the bottom of the hill. In 2006, Takahashi and Yamanaka turned Waddington’s hill upside down by demonstrating that differentiated cells can be reprogrammed into pluripotent stem cells [2]. The creation of induced pluripotent stem cells (iPSCs) is mediated by the induction of four key transcription factors (TF)s: Oct3/4, Sox2, C-Myc and Klf4. This seminal discovery has led to a greater appreciation for the phenotypic plasticity of cells, the ability of committed differentiated cells to change to an alternative committed cell state. Extending Waddington’s metaphor, changes in committed cell state can occur through three general routes, de-differentiation to a less committed multipotent state, de-differentiation and redifferentiation to a different committed state, or direct transdifferentiation between different committed states [3]. These different routes of cell state change have now been documented in multiple tissue types, often in response to tissue damage and injury [4–7]. Phenotypic plasticity is now recognized as an essential component of normal development and tissue homeostasis.

There is a growing body of data demonstrating that, in certain contexts, cancer cells can also exhibit phenotypic plasticity during disease progression and as a means to acquire therapeutic resistance [8,9]. Evidence of cancer cell plasticity has been documented in multiple cancer types [10–14]. This evidence has led to the recognition of phenotypic plasticity as a cancer hallmark [15,16]. The molecular machinery facilitating cancer phenotypic plasticity involves a complex interplay between genetic alterations, lineage-defining TFs, epigenetic regulators, metabolic pathways, and crosstalk within the tumor microenvironment [17–20]. Research within this growing field is complicated further by observations suggesting these interactions are often context dependent. The molecular basis of cancer cell plasticity thus remains incompletely defined.

The definition of phenotypic plasticity remains vague and also context dependent. Whiting *et al*. recently proposed refining the term to mean a change in cellular phenotype induced by environmental stimuli as opposed changes in phenotype that arise stochastically due to transcriptional noise [21]. However, it is often not possible to rule out environmental stimuli in the context of a complex normal or tumor tissue environment. This review, therefore, does not distinguish between stochastic or induced cell state changes, and it defines phenotypic plasticity as the capacity of a cell to transition from one meta-stable phenotypic state to another. This use of phenotypic plasticity also encompasses lineage state changes that are often referred to as lineage plasticity. This review is also focused on transcriptional states as a proxy for phenotype because transcriptional states are heritable through cell division and because the technology for measuring transcriptional states at the single cell level has been available for a sufficient length of time to generate a robust body of published literature. The goal of this review is to highlight convergent molecular pathways that influence cellular plasticity in normal and tumor tissue, to identify key unresolved questions, and to briefly note emerging technologies that may help answer those questions.

## 2. Plasticity in Tissue Homeostasis

### 2.1 Facultative stem cells

**Figure 2.**
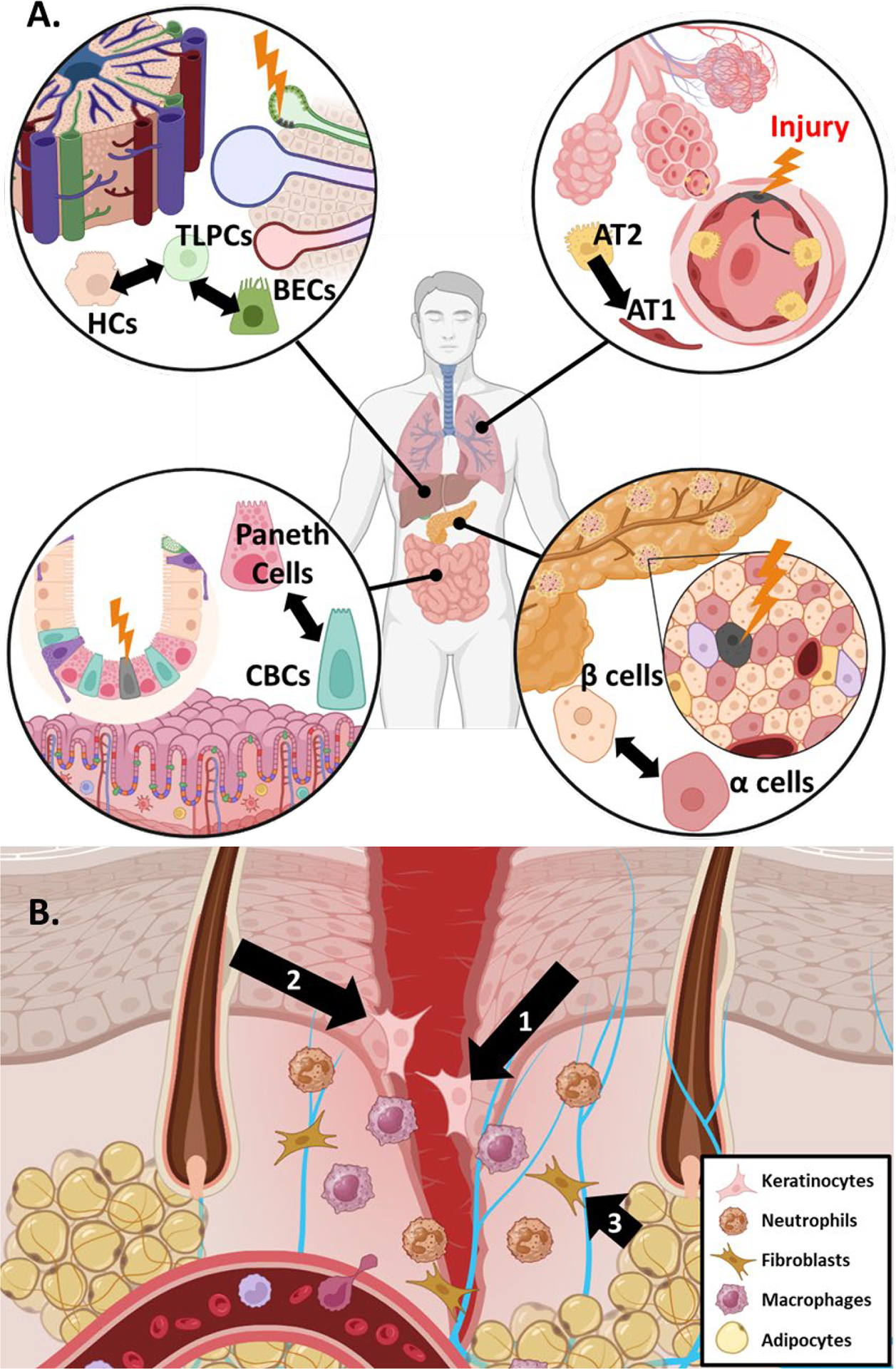
Phenotypic plasticity in normal tissue homeostasis. (**A**) Evidence for phenotypic plasticity and FSC in normal tissue are highlighted by examples in the liver (*top left*), lung (*top right*), intestine (*bottom left*) and pancreas (*bottom right*). When tissue damage occurs, adjacent differentiated cell populations can act as FSCs to replace damaged cell types and maintain homeostasis. (**B**) Evidence for phenotypic plasticity in skin wound healing is depicted including (1) EMP of adjacent keratinocytes, (2) transdifferentiation of cells from the hair follicle niche and (3) transdifferentiation of adipocytes to new myofibroblasts. FSC, facultative stem cell; EMP, epithelial-mesenchymal plasticity; BEC, biliary endothelial cell; TLPC, transitional liver progenitor cells; HC, hepatocyte; CBC, crypt base columnar cells. Created using BioRender.com

Maintaining a balance of multiple cell types is a key part of normal tissue homeostasis. To maintain homeostasis in response to damage, some tissues contain committed differentiated cells that de-differentiate to a less committed multipotent state to assist in the regeneration of damaged cell types (Figure 2) [22]. Such facultative stem cells (FSCs) have unique chromatin dynamics that enable more facile de-differentiation under certain conditions like tissue injury and regeneration [23–25]. Identifying the molecular drivers important for FSC function may yield greater insights into molecular drivers that are also involved in a cancer plasticity discussed below.

Pancreatic islets are small glands comprised of different endocrine cells responsible for regulating blood glucose levels. β cells are the main producers of insulin and the loss of β islet cells is directly associated with the development of diabetes mellitus [26]. In response to injury, it has been demonstrated that multiple other islet cell types act as FSCs to replace lost β cell populations, although the significance of FSC-based repopulation is still debated (Figure 2A) [27,28]. There are many TFs that influence the lineage fate of β islet cells. An extensive review of the various TFs at play in β cell de-differentiation has been published previously [26]. In brief, de-differentiation of β cells is associated with decreased activity of traditional β cell lineage drivers such as PDX1 and NEUROD1 alongside changing activity in stemness associated TFs like Nanog, Hes1 and FoxO1 [29–31]. Previous work suggested that de-differentiation of β cells is also regulated epigenetically by the PRC2 complex, among other epigenetic regulatory complexes [32].

Lung alveoli are an essential epithelial barrier chronically exposed to environmental and pathogenic insults. As a result, homeostasis in this tissue requires robust means to replace damaged alveolar cell types. Alveoli are comprised of two major cell types: alveolar type I (AT1) cells and alveolar type II (AT2) cells [33,34]. AT1 cells account for the majority of alveolar surface area and thus are prone to damage. AT2 cells function to secrete surfactant proteins and maintain fluid balance in the lung, but they can also act as FSCs to replace lost AT1 cells after tissue damage (Figure 2A) [35–37]. Single-cell transcriptomics analysis has revealed that AT2 cell mediated tissue repair occurs in three distinct phases: (1) active cell proliferation (expansion), (2) cell cycle arrest and (3) AT2 to AT1 transdifferentiation [38]. Several signaling pathways have been implicated in coordinating AT2-to-AT1 transdifferentiation including Wnt/β-catenin and Hippo signaling [39–42], pathways that have also been implicated in liver FSC function (below). Kaiser *et al*. recently found that p53 also influences AT2/AT1 cell plasticity [43].

The intestine is another tissue chronically exposed to the environment and is characterized by rapid cell and tissue turnover. LRIG5^+^ adult stem cells at the base of intestinal crypts help support this turnover to maintain homeostasis [44–46]. Crypt base columnar (CBC) stem cells undergo asymmetric division producing one daughter stem cell and one daughter cell destined for differentiation. There remains some debate about the main mechanism contributing to regeneration of lost CBC populations (*i.e*., quiescent reserve stem cells, de-differentiation and, more recently, isthmus progenitor cells) [47]. Regardless, it has been shown previously that various differentiated cells within the intestinal epithelial lining can act to replenish lost CBCs following injury (Figure 2A) [6,48–50]. Plasticity can be partly attributed to rapid epigenetic reprogramming of various secretory and epithelial cells following crypt injury [51]. The plasticity of the intestinal lining is also maintained through a complex intestinal niche that relies on multiple signaling pathways and crosstalk with the immune system. Wnt/β-catenin signaling through Sox9 and Tcf4 has been shown to be essential for the maintenance of CBC stemness [52–54]. Murata *et al*. also identified ASCL2 as an important TF responsible for mediating the replenishment of lost CBCs through de-differentiation [55].

One of the main functions of the liver is to detoxify blood collected from the digestive system, thus the liver has extensive regeneration capabilities to maintain homeostasis during chronic exposure to potential toxicants. The liver’s regenerative capacity is attributed to two cell types: Hepatocytes (HCs) and Biliary Endothelial Cells (BECs) [44,56,57]. There is good evidence to suggest that transdifferentiation between HCs and BECs is a critical component of liver regeneration (Figure 2B) [58–60]. In particular, Pu *et al*. has recently demonstrated the existence of transitional liver progenitor cells which arise from BECs in response to liver injury. Signaling pathways identified as crucial to the plasticity of HCs and BECs include TGF-β, Notch, and Hippo signaling among others [61–63].

### 2.2 Skin wound healing

The skin is the major epithelial barrier to the environment and maintenance of the skin’s integrity is paramount. When the skin is injured, an elaborate dance of epithelial skin cells, immune cells, and multiple signaling pathways is triggered to restore homeostasis. The process of skin wound healing consists of four general phases: hemostasis, inflammation, proliferation and remodeling [64]. These phases occur over different time scales and overlap each other. Excellent reviews on wound healing can be found elsewhere [65,66]. Phenotypic plasticity plays a major role in skin wound healing, particularly the epithelial to mesenchymal transition (EMT). EMT allows epithelial cells to shed many of their intercellular connections (tight junctions, desmosomes, *etc*.) to acquire greater cell motility required for wound healing [67]. EMT has broad impacts on tissue biology beyond the skin, including normal embryonic development [68]. While the term EMT implies two distinct cell states, there is now significant evidence to suggest EMT actually exists as a spectrum of cellular states characterized by varying degrees of epithelial and mesenchymal features [69,70]. This is commonly referred to as Epithelial-Mesenchymal Plasticity (EMP). As part of the wound healing process, keratinocytes at the wound edge need to mobilize and quickly migrate to seal the open wound, beginning the process of re-epithelialization (Figure 2B). To do so, keratinocytes under these conditions activate an EMP program. Previous work shows that deficiencies in the EMP core TF Slug compromises wound closure [71]. The role of other major EMP TFs such as Twist1 and Snail are still under investigation. There is evidence to suggest that endothelial to mesenchymal plasticity (EndMP, a process analogous to EMP) also plays a significant role in mediating wound healing [72,73].

The importance of phenotypic plasticity in wound healing extends beyond EMP and EndMP. The skin houses several unique structures like hair follicles (HF), sebaceous glands, and sweat glands that carry out skin functions beyond barrier integrity. Cells originating within these structures also play an important role in the resolution of wound healing [74]. In particular, cells originating within the HF, the interfollicular epidermis, and other skin cell niches have been shown to act as reservoirs of FSC from which additional keratinocytes can be produced [75–78]. RNA data indicates cells from these different skin niches have distinct transcriptional profiles, but their transcriptional state converges as they assume the FSC phenotype, supporting the existence of substantial plasticity within skin cells [79]. Beyond epithelial cells, Shook *et al*. noted that adipocytes localized to the wound edge also alter their lineage to a myofibroblastic state that contribute to extracellular matrix remodeling (Figure 2B) [80]. However, the validity of this property of adipocytes is still under debate [81]. In a similar vein, Parfejevs *et al*. found that Schwann cells of peripheral glia exhibit significant plasticity, facilitating new growth from damaged nerves within the wound site [82]. These findings highlight the importance of phenotypic plasticity in reepithelialization and remodeling during wound healing.

Many interconnected signaling pathways work together to co-ordinate recruitment of immune cells, keratinocyte proliferation and increased cellular plasticity during skin would healing (Table 1). EMP is driven by multiple signaling pathways including TGFβ, Notch, and Hedgehog signaling [83]. EndMP is driven by many of the same signaling pathways including TGFβ, Notch, and activation of core EMP TFs like Slug and Twist1 [67,73,84–87]. TFs like KLF5 and Sox9 are implicated in enabling plasticity amongst skin cells, with Sox9 playing a major role in promoting partial lineage transitions in multiple skin cell niches [88,89]. To date, transcriptional/epigenetic characterization of wound healing has been limited due to the complexity of analyzing chromatin changes at a single cell resolution. However, recent studies have highlighted that both the proliferative and remodeling phases are characterized by distinct chromatin states within different skin cell niches [89,90]. As single cell analysis approaches expand to include chromatin and spatial information, the major molecular drivers of cellular plasticity in wound healing will likely be revealed in time.

## 3. Plasticity in Neoplasia

Plasticity plays a crucial role in maintaining homeostasis within normal tissues. Plasticity is also exploited in diseased tissue, cancer in particular. Phenotypic plasticity is now recognized as a cancer hallmark [15,16]. Plasticity can endow cancer cells with robust adaptability, a useful trait for advanced cancers that experience unpredictable and highly selective environmental pressures during disease progression. For example, plasticity can arise in response to therapy, providing a means for some cancer cells to adapt and survive therapy to seed acquired resistance. Many molecularly targeted cancer therapies are directed towards molecules that play some role in normal lineage specification and commitment. Inhibiting these molecules in sufficiently plastic cancer cells may induce or select for loss of differentiation. In extreme cases, such treated cancer cells may reprogram into an alternative lineage state no longer dependent on the therapeutic target, rendering those cells resistant. Current evidence suggests cancer plasticity may increase as more potent molecularly targeted cancer therapies are deployed in the clinic [91]. Treatment-associated phenotypic plasticity has now been documented in several different cancer types (Table 2). Select examples are reviewed below.

### 3.1 Prostate cancer

Treatment for metastatic prostate adenocarcinoma (PCa) relies on targeting androgen receptor (AR) signaling that PCa depends on for growth and viability. Androgen-deprivation therapy (ADT) reduces the levels of circulating androgens that serve as activating ligands for AR. Androgen receptor signaling inhibitors (ARSIs) like enzalutamide target AR itself to block its activation. ADT and ARSIs are effective in treating most PCa patients, but they are not curative. While multiple genetic mechanisms mediate acquired therapeutic resistance, up to 20–25% of PCa progressing through AR targeted therapies show evidence of phenotypic plasticity, in particular transdifferentiation to neuroendocrine prostate cancer (NEPC) lineage variants [127–129]. NEPC is highly aggressive and NEPC patient prognosis is dismal because effective therapies are not available. Development of NEPC has been linked to the loss of tumor suppressor genes such as RB1, TP53 and PTEN [92]. More recently, other key NEPC drivers have also been identified including ASCL1 and N-Myc [103,130,131]. AR independent lineage variants that lack neuroendocrine differentiation, referred to as double negative prostate cancer (DNPC), have also been detected clinically. Recent work has identified several key TFs like Sox2, FOXA2, ZNF263 and KLF5 that drive DNPC [104–106].

### 3.2 Lung cancer

Mutations in the epidermal growth factor receptor (EGFR) gene account for about 27% of lung adenocarcinoma (LUAD) cases [132,133]. Oncogenic mutations in EGFR lead to constitutive activation of this receptor kinase which drives increased cell proliferation and survival [134]. EGFR tyrosine-kinase inhibitors (EGFR TKIs) are a suite of drugs designed to target EGFR tyrosine kinase activity. First line therapy for patients with EGFR-mutant LUAD is currently treatment with the third generation EGFR TKI Osimertinib [135]. While an effective therapy, virtually all patients eventually acquire resistance through multiple on- and off-target mechanisms (bypass mutations, additional EGFR mutations, *etc*.) [136]. In a smaller subset of cases, resistance to Osimertinib has been linked to phenotypic plasticity. In these patients, therapeutic resistance is associated with reprogramming of LUAD cells into neuroendocrine lineage variants similar to de novo small cell lung cancer (SCLC) [14,108,137]. As in PCa, these treatments associated neuroendocrine lineage variants exhibit recurrent mutations in the RB1, TP53, and PTEN tumor suppressor genes [14,107]. Furthermore, ASCL1, Sox2 and Myc have been identified as possible drivers of neuroendocrine plasticity in LUAD [96,99,115]. Additional lung cancer lineage variants identified include adeno-to-squamous and adeno-to-mesenchymal variants [110,113,116]. Loss of functional LBK1 has been directly associated with adeno-to-squamous transdifferentiation (AST) [109,114]. Myc, AKT and JAK/STAT signaling have also been implicated in facilitating AST [110,111]. Hu *et al*. demonstrated that the neuroendocrine TF ASCL1 leads to Osimertinib resistance by promoting a more permissive cellular chromatin state and supporting an EMP-gene expression program [115]. Recent evidence suggests that EMP-based cell states act as intermediate stepping stones to other variant lineage states [138]. ASCL1 promotion of an EMP-gene program may thus be an initial step towards neuroendocrine reprogramming. Work by Hu *et al*. and others also highlighted the importance of altered chromatin dynamics in mediating phenotypic plasticity in both PCa and LUAD [97,101,115,139–141].

### 3.3 Other cancers

Some of the earliest evidence for cancer phenotypic plasticity has come from the study of patients with melanoma. Advanced melanoma cells can exhibit a phenotype that mimics endothelial cells in blood vessels, expressing key endothelial cell specific genes and contributing to tumor vascularization. This phenomenon is termed ‘vasculogenic mimicry’ (VM) and has since been observed in other cancers as well [12,117]. Recent studies suggest β-catenin signaling through Tcf-4 drives melanoma VM through increased expression of EMP and pluripotency TFs [118]. Triple negative breast cancer (TNBC) is characterized by the absence of HER2, estrogen receptor, and progesterone receptor expression. Certain subsets of TNBC display remarkable capacity for lineage infidelity. For example, metaplastic breast cancer often exhibits phenotypic plasticity in the form of EMP and increased stemness [142]. More recently, Stevens *et al*. identified a luminal-to-mesenchymal/basal lineage shift in inflammatory breast cancer (IBC) facilitated by JAK-STAT signaling and Sox10 [119,143]. In neuroblastoma, adrenergic-mesenchymal transdifferentiation was found to be dependent on the SWI/SNF chromatin remodeling complex [121]. Xu *et al*. demonstrated that targeting ATPases in the SWI/SNF complex causes displacement of several core TFs including N-Myc. This suggests a permissive chromatin state allows for elaboration of new transcriptional programs to expand phenotypic plasticity. Pancreatic Ductal Adenocarcinoma (PDAC) is known for its considerable heterogeneity that makes it refractory to treatment. Work by Murakami *et al*. has identified rare PDAC cells marked by reactivation of pluripotent TFs, and this reactivation is coupled to increased EMP [122]. These stem-like PDAC cells are less dependent of oncogenic Yap signaling, suggesting that increased stemness and plasticity in a subset of PDAC cells may contribute to disease progression in the presence of therapy. Hepatocellular carcinoma (HCC) stem cells have previously been implicated in therapeutic resistance [144]. More recent work suggests that HCC cancer stem cells arise through de-differentiation of bulk HCC tumor cells and that this de-differentiation is dependent on the transcriptional activity of SPINK1 [123]. In bladder cancer, Warrick *et al*. determined that urothelial and squamous cell states are derived from a common precursor [124]. They also identify FOXA1 as a critical lineage TF maintaining lineage fidelity. Loss of FOXA1 was found to promote epigenetic reprogramming leading to the squamous phenotype, supporting the idea that phenotypic plasticity contributes to heterogeneity in bladder cancer.

In summary, phenotypic plasticity and lineage infidelity have clear and widespread roles in cancer progression and therapeutic resistance (Table 2). As a relatively new cancer hallmark that has only come under intense study recently, additional examples of cancer phenotypic plasticity are likely to be found in the future. One reason for this is the increasing deployment of single cell technologies that can measure heterogeneity between cancer cells within a given tumor, potentially over time and space. These technologies are likely to uncover new phenotypic cancer cell variants that would otherwise go undetected by routine analysis of tissue section histopathology, particularly if they only exist transiently. For example, phenotypic plasticity is inherent in the drug tolerant persistor (DTP) cells that have been described in numerous experimental models of cancer. DTPs are rare cells within a homogeneous cancer cell population that survive therapy while the bulk of the cancer cells don’t. While these cells exist transiently, they are required to facilitate acquired therapeutic resistance by both genetic and non-genetic mechanisms. Transient changes in phenotypic states have also been detected by single cell approaches in patient tumors as they progress from residual disease to progressive disease, mirroring dynamic phenotypic plasticity in experimental models [145]. For a more in-depth exploration of the DTP field, see the review from Russo *et al*. [146]. As single cell technologies expand further to measure an increasingly broader array of molecular changes, dramatic advances in our understanding of the molecular basis of phenotypic plasticity are likely to be made.

## 4. Molecular Determinants of Cancer Plasticity

Although a great deal has been learned about the molecular mechanisms influencing cancer phenotypic plasticity, there remain many unanswered questions. Chief among them is which molecular pathways control the extent of plasticity itself, as opposed to the pathways that direct the phenotype/lineage that plastic cells are likely to assume. In the context of cancer this is an important question if the goal is to manipulate or target cancer phenotypic plasticity for durable therapeutic benefit. The following section is a summary of molecular pathways that have been implicated in cancer phenotypic plasticity.

### 4.1 Loss of tumor suppressor genes

**Figure 3.**
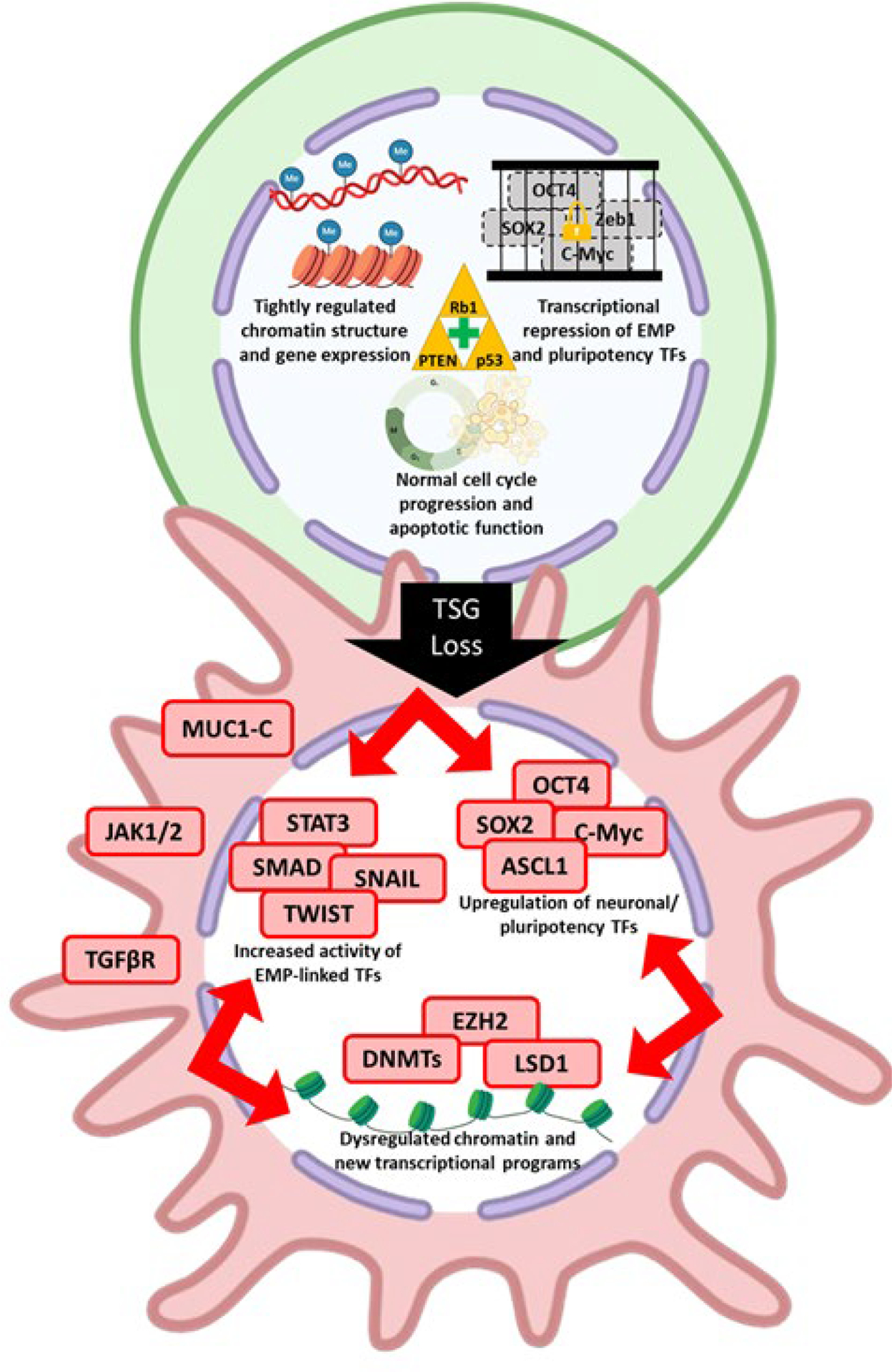
Molecular determinants of cancer phenotypic plasticity. Phenotypic reprogramming of cancer cells is associated with dramatic transcriptional changes, often following the loss of key TSGs. The top cell shows a non-plastic, stable cancer cell state in which functional TSGs maintain transcriptional stability. The bottom cell shows a TSG-deficient cancer cell with heightened phenotypic plasticity. Such cells are sensitized to the effects of increased lineage specific TF activity, like EMP and pluripotency TFs. These TF can now drive the cancer cell to a new phenotypic state. Progressive evolution of phenotypic states promotes increasing invasiveness and treatment resistance. TSG, tumor suppressor gene; TF, transcription factor; EMP, epithelial-mesenchymal plasticity. Created using Biorender.com

Tumor suppressor genes (TSGs) have long been known to play a fundamental role in protecting cellular integrity and mitigating dysregulated cell proliferation. Of the known TSGs, some appear to have context specific roles while others have widespread roles across many cancer types. Among the many molecular aberrations that have been linked to phenotypic plasticity, loss of key TSG functions is one of the most recurrent across different solid cancers (Table 2, Figure 3).

*TP53* encodes the protein p53, a TF whose main function is to regulate the cell cycle and programmed cell death in response to various stresses, including DNA damage. In this respect, p53 is considered the “guardian of the genome” [147]. There is significant evidence to suggest, however, that the loss of functional p53 also promotes increased lineage infidelity in multiple cancer types. In both PCa and LUAD, loss of p53 is associated with lineage reprogramming [14,92,108]. Notably, p53 plays an important role in regulating the activity of pluripotent TFs, such as Nanog and Sox2, and in regulating DNA methylation of the genome [148]. In the absence of p53, increased activity of pluripotency TFs and dysregulated DNA methylation contribute to greater phenotypic plasticity [148]. p53-mediated regulation of pluripotency TFs is further supported by prior evidence indicating p53 restricts reprogramming of iPSCs [149]. Previous work from Ku *et al*. shows a clear role for p53 loss in amplifying PCa plasticity [92]. Prostate specific deletion of *Pten* and *Rb1* in the mouse PCa that responds to castration, but relapses as NEPC with acquisition of spontaneous inactivating *Trp53* mutations. Engineered mutations of *Pten*, *Rb1* and *Trp53* cause PCa that rapidly progresses to NEPC. Analogous findings are observed with similarly engineered human PCa cell lines [92,95]. More recent work by Chan *et al*. highlights additional lineage states that develop in these mice beyond NEPC [93]. As mentioned previously, p53 has also been implicated in AT2-to-AT1 transdifferentiation during alveolar injury repair [43]. Kaiser *et al*. also demonstrated that when p53 is lost, there is subsequent accumulation of cells with a hybrid AT2-AT1 cancer cell state. This data, alongside earlier evidence from the literature, support a role for p53 in regulating cancer phenotypic plasticity in addition to its canonical roles as a stress responsive regulator of the cell cycle and programmed cell death [150,151].

The TSG *RB1* encodes a transcriptional repressor protein Rb1 and is mutated recurrently across a wide variety of cancer types. Rb1’s canonical function is cell cycle regulation. Rb1 binds to the E2F family of TFs, switching them from transcriptional activators to transcriptional repressors, and blocking the expression of genes required for the cell cycle. This function is mediated by Rb1’s ability to recruit histone deacetylases (HDACs), histone methyltransferases (HMTs), and DNA methyltransferase (DNMTs) to E2F target genes and altering chromatin near these genes to more transcriptionally repressive states [152]. Rb1, like p53, has a variety of additional non-canonical functions that have been reviewed elsewhere [153,154] including a role in cancer phenotypic plasticity. Rb1 loss of function mutations are highly recurrent in treatment associated neuroendocrine cancers that arise through reprogramming of PCa and LUAD tumors [14,92,108]. Multiple Rb1 molecular functions potentially explain why loss of function drives increased plasticity. Rb1 is a central epigenetic hub interacting with many complexes that regulate chromatin structure (e.g., SUV39H1, EZH2, *etc*., reviewed by Guzman *et al*.) [152,155]. Rb1 has been shown to maintain both constitutive heterochromatin (e.g., pericentric and telomeric regions, repetitive elements) and facultative heterochromatin [156–159]. Since the epigenome must be reconstituted after every S phase of the cell division cycle, loss of Rb1-mediated cell cycle control may also destabilize the epigenome. Sanidas *et al*. demonstrated that Rb1 interacts with chromatin insulators like CTCF to facilitate chromatin organization [160]. Rb1 is also known to repress pluripotency signaling networks [161]. Widespread dysregulation of chromatin organization and increased activity of pluripotency TFs (e.g., Sox2) in the wake of Rb1 loss are likely major contributors driving phenotypic plasticity in PCa and LUAD [95]. Dean *et al*. previously showed that Rb1 is responsible for repressing the activity of the EMP TF Zeb1 in invasive LUAD [162]. Increased Zeb1 activity following Rb1 loss, promoted by a positive feedback loop between Ets1 and Zeb1, likely plays a role in exacerbating EMP in LUAD. Given recent implications about the contribution of EMP in the progression to different cell states, Rb1-mediated repression of EMP may be an important safeguard against broader plasticity [138].

*PTEN* encodes a phosphatidylinositol-3,4,5-trisphosphate 3-phosphatase whose canonical function is to negatively regulate the PI3K/AKT signaling pathway [163,164]. PI3K phosphorylates phosphatidylinositol bisphosphate (PIP2) to phosphatidylinositol-3,4,5-triphosphate (PIP3), and PIP3 subsequently facilitates activating phosphorylation of AKT, a major intracellular protein kinase that regulates cell growth. PTEN is a phosphatase that acts in opposition to PI3K, catalyzing the conversion of active PIP3 to inactive PIP2. In doing so, PTEN suppresses continuous growth and survival signaling facilitated by active AKT. Loss of function PTEN mutations are recurrent in many different types of cancer [165,166]. Pertinent to this review, PTEN loss has more recently been shown to promote greater phenotypic plasticity. Zhang *et al*. has found that *Pten* knockout amplifies *in vivo* tumor heterogeneity of LUAD developing in genetically engineered mice lacking functional p53 and Rb1 [107]. Transcriptomic profiling of genetically engineered mouse model (GEMM) lung tumors deficient for *Pten* revealed greater inter- and intra-tumoral heterogeneity, suggesting PTEN plays a role in suppressing phenotypic plasticity. Work from another group implicates PTEN in regulating the plasticity of hematopoietic lineages and progression to leukemia [167]. Xu *et al*. demonstrated that PTEN enforces B-cell lineages by negatively regulating the activity of PU.1, a TF known to regulate chromatin accessibility during the course of hematopoiesis [168]. PTEN-null mice exhibit defective B-cell lineage differentiation and greater T- and Myeloid-lineage differentiation which was found to be mediated through PU.1-altered chromatin accessibility. Most importantly, Xu and colleagues identify PTEN-null prepro-B cells as a potential precursor to T-lineage acute lymphoblastic leukemia, highlighting the significance of PTEN-mediated lineage fidelity in tumor suppression. The study also underlines the importance of PTEN’s non-canonical roles in guarding against plasticity. PTEN localized to the nucleus has been demonstrated to facilitate chromatin condensation (through interactions with histone H1), participate in DNA damage repair, and promote centromere stability (see Yang and Yin for review) [164]. PTEN is also notable in that it forms a positive feedback loop with p53 whereby PTEN stabilizes p53 and p53 TF activity promotes increased production of PTEN [169,170]. Finally, PTEN activity is negatively regulated by core EMP TFs such as Zeb1 and Snail1 [163]. Considering PTEN’s role in suppressing plasticity, down regulation of PTEN activity by EMP suggest PTEN loss is a potent catalyst for greater plasticity.

### 4.2 Transcription factors

The loss of TSGs appears to be a major factor influencing phenotypic plasticity in cancer cells, but not all cancers lacking these TSGs undergo detectable phenotypic or lineage state changes. This suggests loss of these TSG may enable increased plasticity, but they may not directly determine the phenotype or lineage state these plastic cancer cells assume. There is extensive evidence to suggest that key lineage promoting TFs activate transcriptional programs that determine cell state changes and/or lineage stability in the context of phenotypic plasticity (Table 2).

The Myc family of TFs are well established oncogenes. The family consists of three members: MYC (C-Myc), MYCN (N-Myc) and MYCL (L-Myc). In depth reviews on the history of the Myc family and its functions can be found elsewhere [171,172]. In brief, Myc TFs play an important role in assembling multi-protein complexes at multiple stages of the transcription cycle suggesting Myc TFs are transcriptional amplifiers. Upregulation of Myc TFs can promote high transcriptional activity that many cancers rely upon for their growth and proliferation [173,174]. More recent literature suggests Myc TFs are important regulators of phenotypic plasticity. Berger and colleagues have previously shown that N-Myc promotes significant transcriptomic and epigenetic rewiring in PCa that contributes to NEPC reprogramming [100]. In the therapeutic context of castration, N-Myc binds at neural lineage genes, alters bivalent H3K4me3 and H3K27me3 histone marks, and promotes expression of neural lineage genes. Berger *et al*. also demonstrated functional divergence between C-Myc and N-Myc within PCa, suggesting individual members of the Myc family may have distinct roles during cancer progression. Work by Wei *et al*. further cements the role of Myc TFs in controlling chromatin dynamics through interactions with the key structural organizer CTCF. C-Myc significantly repressed the expression of neuroendocrine lineage genes via disruption of enhancer-promoter chromatin looping [175]. This finding is corroborated by the observation from Berger *et al*., that C-Myc expression decreased as PCa progresses towards NEPC. However, another study has demonstrated that overexpression of both C-Myc and AKT in normal prostate epithelial cells is sufficient to induce neuroendocrine differentiation [176]. Furthermore, Quintanal-Villalonga *et al*. recently has shown that proteasomal degradation of C-Myc by CDC7 inhibits neuroendocrine differentiation in LUAD and PCa [99]. The conflicting roles of C-Myc in promoting or hindering neuroendocrine differentiation requires further study. Recent work by Gardner *et al*. demonstrates how C-Myc upregulation promotes reprogramming of AT2 cells to a neuroendocrine phenotype [177]. However, C-Myc is not sufficient for neuroendocrine reprogramming because concomitant deletion of p53, Rb1 and PTEN is required. This study highlights the co-operative interactions between TSG loss and TFs like C-Myc in promoting cancer phenotypic plasticity, with TSG loss potentially enabling greater transcriptional heterogeneity that sensitizes cells to the effects of increased C-Myc activity. Similar roles for C-Myc have been identified in other lineage variants (e.g., AST) and in other cancer types (e.g., liver, PDAC) [11,110,178].

Another major TF that has been connected to phenotypic plasticity is Sox2. Like C-Myc, Sox2 is one of the core TFs that together can reprogram differentiated cells to iPSCs [2]. Sox2 plays a major role in normal development, but can also drive stemness, EMP and chemoresistance in cancer [179,180]. Increased Sox2 expression in the context of Rb1 and p53 loss has been previously linked to neuroendocrine lineage reprogramming in PCa [95]. Importantly, Sox2 is known to be a pioneer TF, a class of TF that can alter chromatin accessibility to facilitate new transcriptional programs (reviewed in Hagey *et al*.) [181]. As such, increased Sox2 activity could open previously inaccessible chromatin to enable a wider range of potential transcriptional programs, perhaps accounting for its role in iPSC reprogramming. In this case, SOX2 can be thought of as a stem-like lineage specifying transcription factor that directly enables phenotypic plasticity. Given the role of SOX2 in neuronal lineage specification, it may also function to influence the phenotype of plastic cells. Consistent with this potential role, inhibition of the nuclear transport protein Exportin 1 decreases Sox2 protein levels and inhibits neuroendocrine reprogramming [96].

ASCL1 is an important neuronal lineage specifying pioneer TF during normal development [182]. ASCL1 has also been implicated in the neuroendocrine reprogramming of PCa cells [183] and in defining SCLC subtypes [184]. More recent evidence suggests ASCL1 TF activity is functionally required for neuroendocrine reprogramming of cancer cells. Nouruzi *et al*. has demonstrated that suppressing ASCL1 expression reverses neuroendocrine differentiation; this reversal was linked to chromatin alterations after loss of EZH2 activity downstream of the UHFR1/AMPK axis [185]. Work from two separate groups indicate that abrogation of ASCL1 significantly hinders the progression of PCa to NEPC [94,103]. Rodarte *et al*. noted that tumors with genetic deletion of ASCL1 do not undergo neuroendocrine reprogramming, but instead assume a more basal epithelial phenotype. Interestingly, Romero *et al*. found that ablation of ASCL1 in organoids prior to implantation yields adenocarcinoma tumors with increased AR activity. Conversely, ablation of ASCL1 in developed NEPC tumors led to a modest but temporary tumor regression. Evidence from these papers suggest ASCL1 is a functional driver of neuroendocrine differentiation in NEPC. As mentioned earlier, Hu and colleagues have indicated that ASCL1 drives plasticity in EGFR-mutant LUAD by increasing chromatin accessibility at core EMP TFs like Zeb1 [115]. ASCL1 activity is upregulated during therapy, but a mechanism linking treatment to increased ASCL1 has not been identified definitively. One potential mechanism identified in PCa suggests therapy induces ROR2 signaling that upregulates ASCL1 expression through CREB signaling [186]. Whether a similar mechanism drives ASCL1 expression in LUAD remains to be determined. These observations highlight the role of ASCL1 in regulating both transcription and chromatin to effect phenotypic plasticity.

A number of other lineage-defining TFs have been implicated in reprogramming of cancer cells to alternative phenotypic and lineage states (Table 2). Lineage TF FOXA2 is known to drive neuroendocrine differentiation in PCa, both through promotion of KIT pathway activity and chromatin rewiring in collaboration with JUN [97,98]. FOXA2 and FOXA1 have been shown to promote phenotypic plasticity in NKX2–1^+^ LUAD [187,188]. ONECUT2 is functionally linked to phenotypic plasticity in both PCa and breast cancer [101,120]. AST plasticity has been connected to the increased activity of the TF p63 in both LUAD and PDAC [112,114,189,190]. Zeb1/2, Snail, Slug and Twist are core EMP TFs that can drive phenotypic plasticity and therapeutic resistance [70]. Several studies support the notion that EMP TFs contribute to an intermediate, high plasticity state that can subsequently evolve beyond EMP to assume other lineage states like neuroendocrine [13,191,192].

### 4.3 Signaling pathways

The Janus kinase (JAK) / signal transduction and transcription activation (STAT) signaling axis is a major molecular signaling pathway responsible for many important functions including regulation of inflammation and immunity. IL-6/JAK/STAT3 signaling has also been implicated in tumorigenesis for its impact on chronic inflammation, metastasis, and immune suppression [193]. Recent evidence suggests JAK/STAT signaling also plays an important role in modulating cancer phenotypic plasticity. In PCa, JAK/STAT activation increases during intermediate stages of lineage reprogramming [93,194]. Furthermore, inhibition of JAK and fibroblast growth factor receptor (FGFR) increases AR expression, decreases expression of alternative lineage markers like vimentin, and re-sensitizes PCa cells to ARSIs [93]. Notably, JAK/STAT activity declines once PCa cells undergo reprogramming to a neuroendocrine phenotype, suggesting JAK/STAT activation coincides with development of a high plasticity intermediate state that enables reprogramming to alternative lineages. In lung cancer, JAK/STAT activation in ELM4-ALK condensates is essential for mediating the AST [111]. Inhibition of JAK activity decreases expression of squamous lineage markers and increases the sensitivity of ALK-driven tumors to lorlatinib. Specifically, STAT3 activation is responsible for driving AST in EML4-ALK driven lung cancer. A JAK/STAT dependent cell state has also recently been identified in IBC [119]. Phospho-STAT3 ChIP sequencing of paclitaxel/doxorubicin resistant IBC lines shows a significant enrichment of EMP-related genes including ZEB2 and BCL3. Furthermore, treatment-resistant IBC cell lines express a mesenchymal transcriptional program indicative of a luminal to mesenchymal switch. Progression to this mesenchymal cell state is hindered both by JAK inhibition alone and combined with chemotherapy [119]. JAK/STAT signaling also promotes increased stemness following the inactivation of Notch signaling in oral cancer cells [195]. In gastric cancer, JAK/STAT signaling has been identified as an important driver of ADAR1-based RNA editing which is itself associated with greater chemotherapeutic resistance and stemness [196]. Leukemia Inhibitory Factor (LIF) has been shown recently to inhibit gastric cancer EMP and stemness in a JAK/STAT dependent manner [197]. Intriguingly, Chan *et al*. found that the LIF-LIFR ligand-receptor pair is one of the most enriched in PCa organoids exhibiting phenotypic plasticity. These observations indicate inflammatory signaling, particularly JAK/STAT activation, is recurrently associated with phenotypic plasticity across a range of different cancers. How JAK/STAT activation enables this transcriptional plasticity is not entirely understood.

Transforming growth factor beta (TGF-β) is a potent cytokine that regulates early development, induces plasticity through EMP, and can have major effects on the immune system [198]. TGF-β is known to play a crucial role in normal wound healing, suppressing initial inflammation and mediating the repair of injured tissue [199,200]. TGF-β signaling also impacts numerous cancer types including those arising in the pancreas, liver, breast, and lung among others [201–203]. Some work suggests a direct role for TGF-β signaling in a phenotypic plasticity. TGF-β signaling silences the expression of the histone methyltransferase KMT2D via the upregulation of miR-147b [204]. Lu *et al*. has found that loss of KMT2D expression leads to increased production of activin A, another member of the TGF-β cytokine family. Activin A activates non-canonical p38-MAPK signaling, promoting pancreatic tumor cell plasticity and greater metastatic potential. Thus, multiple members of the TGF-β cytokine superfamily may influence phenotypic plasticity. Blockade of TGF-β in pancreatic cancer suppresses the basal phenotypic state in favor of the classical phenotypic state [205]. In breast cancer, TGF-β works in tangent with AURKA to mediate increased activity of ALDH1, a known marker of stemness [206–208]. SNAI1 expression downstream of TGF-β and AURKA activity induces ALDH1, promoting greater chemoresistance and increased capacity for self-renewal. Fan *et al*. determined that TGF-β signaling is negatively regulated by lncRNA LITATS1-mediated proteasomal degradation of type I TGF-β receptors [209]. Additionally, when LITATS1 is depleted, a dramatic increase in invasiveness and EMP ensues, suggesting TGF-β signaling is driving greater plasticity. In PCa, loss of FOXA1 leads to increased TGF-β3 production, promoting EMP and a more basal-like lineage state [210,211]. However, another group has demonstrated that prostate tumors lacking the TGF-β type II receptor (Tgfbr2) express increased levels of stem cell and basal epithelial marker genes [212]. Furthermore, mutations in PTEN and Tgfbr2 lead to an increase in metastatic burden, suggesting that loss of TGF-β signaling does not hinder EMP in the PCa setting. These studies suggest a more complex and potentially context dependent role for TGF-β signaling in PCa plasticity that requires further study.

Notch signaling is a major architect of development and repair and its numerous functions have been reviewed elsewhere [213]. Notch signaling plays an important role in plasticity-mediated repair of several normal tissue types including lung alveoli and intestinal crypts [214,215]. Recent evidence indicates a role for Notch signaling in cancer lineage reprogramming. Ku *et al*. found that Notch signaling is downregulated during reprogramming of PCa to NEPC [216]. Furthermore, forced Notch signaling suppressed NEPC development while maintaining PCa tumors in a more inflammatory state with significant effects on the tumor immune microenvironment. This is potentially consistent with the known roles of Notch signaling in the immune system [217]. These observations suggest the lineage state assumed by plastic cancer cells may significantly impact the tumor microenvironment. Similar findings have been made in breast cancer showing that Notch signaling influences cancer phenotypic plasticity [218]. In this case, FRMD3 mediated activation of Notch signaling promotes basal-to-luminal differentiation. Consequently, loss of FRMD3 in mammary epithelial cells leads to decreased Notch signaling and increasing stemness that promotes TNBC development.

### 4.4 Other mediators of plasticity

DNMTs and HMTs are the major enzymatic writers of DNA and histone modifications, respectively, that regulate chromatin structure and accessibility. Several of these epigenetic regulatory enzymes are implicated in phenotypic plasticity. EZH2 encodes the major catalytic protein of the PRC2 complex that tri-methylates histone H3 at K27, a chromatin mark associated with transcriptional repression. This complex has been prominently implicated in cancer progression (for review see Liu and Yang) [219]. EZH2 mediated regulation of chromatin accessibility has also been implicated in phenotypic plasticity. Berger *et al*. shows that EZH2-inhibition can rescue luminal gene expression that has been suppressed by N-Myc activity [100]. EZH2 has also been shown to interact with ASCL1 to promote chromatin remodeling [185]. Recent work from Venkadakrishnan and colleagues show many TFs associated with neuroendocrine differentiation, such as ASCL1 and NEUROD1, are silenced via H3K27me3 in PCa but not in NEPC. Furthermore, EZH2 inhibition upregulates NEPC TF activity at promoters that are normally bivalent in the absence of EZH2 inhibition [220]. Modulation of EZH2 activity through phosphorylation is an important means of regulating EZH2-associated plasticity, highlighted in work by Nouruzi *et al*. [18,185]. A mechanism linking ADT and increased EZH2 activity has been described by Kaarijärvi *et al*. [221]. DPYSL5, a protein involved in neuronal differentiation, is actively suppressed by androgen signaling. When androgen signaling is lost, increased DPYSL5 promotes increased EZH2 activity and subsequent upregulation of NEPC-linked TFs like ASCL1 and Sox2. Contrasting roles for EZH2 in inhibiting or promoting NEPC TFs proposed by Venkadakrishnan *et al*. and Kaarijärvi *et al*., respectively, suggest a nuanced role for EZH2 in progression towards NEPC that requires further study.

LSD1 (also known as KDM1A) is a well-known histone demethylase that has been implicated in cancer phenotypic plasticity [222]. Han *et al*. has demonstrated that LSD1 activity is essential for full elaboration of the E2F1 mediated transcriptional program in Rb1 deficient PCa [223]. This study implicates LSD1 as an important regulator of chromatin interactions in the progression from PCa to NEPC. FOXA2-driven plasticity in AR-independent PCa is also dependent on LSD1 activity [97]. Mandl *et al*. recently has shown that LSD1 inhibition decreases ASCL1 dependent transcriptional activity through de-repression of YAP1. This suggests functional LSD1 is important for the ability of ASCL1 to promote neuroendocrine differentiation [224]. LSD1 activity has also been found to mediate plasticity in breast cancer [225,226]. In general, the effectiveness of LSD1 inhibitors in treating cancers seems to correlate with their plasticity [227].

DNMT1 is an important DNMT that is responsible for maintenance of methylation during DNA replication of the genome [228]. DNMT1 activity is perhaps even more important for the transcriptional stability of cancer cells undergoing continuous cell division cycles. Expression of DNMT1 often increases during cancer progression, including during the transition from PCa to NEPC. DNMT1 has also been implicated functionally in PCa phenotypic plasticity, suggesting that targeting DNMT1 activity and the aberrant DNA methylation it produces is a potential therapeutic strategy for treating NEPC [229,230]. DNMT1 activity has also been functionally implicated in breast and pancreatic cancer plasticity [231–233].

Mucin-1 (MUC1-C) is a transmembrane cell surface protein that plays an important role in maintaining the mucosal barrier of epithelial cells. Aberrant glycosylation of MUC1-C in cancer has been implicated in cancer invasion and metastasis [234]. Evidence from the literature suggest that MUC1-C can play an important role in neuroendocrine plasticity [102,235]. MUC1-C suppresses p53 activity, promotes activity of pluripotent TFs downstream of BRN2, and upregulates the activity of the Baf chromatin remodeling complex [235–237]. MUC1-C has recently been implicated in Osimertinib resistance, promoting EMP and proliferative signaling in EGFR-mutant NSCLC [238]. MUC1-C also promotes plasticity in TNBC [239,240]. A recent review highlights that chronic activation of MUC1-C in wound healing is an impetus for epigenetic rewiring and progression towards cancer [241]. Given that MUC1-C is a surface protein, MUC1-C may be a useful target for suppressing cancer phenotypic plasticity through a number of immunotherapeutic approaches currently in development.

## 5. Discussion

This review highlights the extensive crosstalk that exists between genetic alterations, TFs, signaling pathways (cancer cell intrinsic and extrinsic), and epigenetic regulators that complicate our understanding of cancer phenotypic plasticity (Figure 3). Despite this complexity, a comparison of pathways involved in normal and neoplastic tissue reveals some convergence. The TF ASCL1 plays a direct role in maintenance of CBC cells in intestinal crypts during normal homeostasis and is also important for neuroendocrine lineage reprogramming in several cancer types. Notch signaling modulates plasticity to facilitate repair of damaged normal tissues while also suppressing neuroendocrine reprogramming in cancer. Major pluripotency TFs like Sox2 are important for facultative stem cell plasticity in normal tissue while also driving cancer phenotypic plasticity. Inflammatory signaling is involved in normal cell plasticity during tissue repair and has also been functionally linked to phenotypic plasticity in multiple cancer contexts. MUC1-C activity connects plasticity in normal wound healing with cancer phenotypic plasticity, recapitulating the idea that cancer is a “wound that does not heal” [242]. This convergence highlights the notion that cancer cells use genetically programmed plasticity programs in aberrant ways to adapt to the unpredictable and highly selective pressures they face.

One major difference between normal and neoplastic cell plasticity is that cancer cells frequently acquire somatic genetic alterations. This raises the possibility that some genetic alterations recurrent in advanced cancers may be selected because they facilitate aberrant activation of phenotypic plasticity programs. Using Waddington’s metaphor, phenotypic plasticity associated genetic alterations can be classified in two non-mutually exclusive categories. Some mutations may flatten the epigenetic landscape overall, driving increased phenotypic plasticity by making transitions between different phenotypic states more likely. Other genetic alterations may change the landscape by increasing or decreasing the depth of valleys specifying individual phenotypic states, decreasing or increasing the probability a cancer cell will assume a specific phenotypic state. Loss of TSGs like RB1, TP53, and PTEN seem to fall in the first category. Clinical data indicate loss of these TSGs are highly recurrent in treatment associated neuroendocrine cancer lineage variants, but TSG loss is not sufficient because not all affected cancers undergo detectable lineage reprogramming. Further, loss of these TSG in GEMMs of prostate cancer develop adenocarcinomas along with rare cells undergoing reprogramming into multiple lineage variants, including NEPC that expands the fastest to yield lethal disease. Other genetic alterations may fall closer to the second category. C-Myc, for example, can drive SCLC to a unique lineage state, but only in collaboration with plasticity afforded by TSG loss [243,244].

A number of incompletely resolved questions remain for the cancer phenotypic plasticity field. Related to the discussion above, one question is how do genetic alterations recurrent in advanced cancer actually contribute to phenotypic plasticity at the molecular level. Phenotypic plasticity is defined here by transcriptional heterogeneity and instability. The molecular basis of this transcriptional heterogeneity is not well defined, but by analogy to observations with iPSCs may involve an increase in bivalent, stem cell-like chromatin state. Another unresolved question is how inflammatory signaling drives phenotypic plasticity. Loss of TSGs often result in elevated cancer cell intrinsic inflammatory signaling, so it is possible effects on TSG loss in phenotypic plasticity are mediated by inflammatory signaling, at least in part. Currently, it is unclear how this inflammatory signaling causes the transcriptional instability associated with phenotypic plasticity. Another important question yet to be resolved is the relationship between cancer phenotypic variants detected in both clinical samples and experimental models. Do these different variants represent different stages of progression across a common, lineage phenotypic evolutionary path? In some experimental models, there is evidence to support this conclusion. Or do they represent independent evolutionary paths that may yield cancers with very different clinical outcomes? The answers to these questions will help clarify the molecular basis of cancer phenotypic plasticity, information that will be critical for developing therapeutic approaches to effectively target phenotypic plasticity for clinical benefit.

Robust quantitative measures of phenotypic plasticity are needed to resolve these questions. An ideal measure would monitor the phenotype of individual live cells over time in an unbiased manner, quantitating how stable a phenotype is to stochastic fluctuation or environmental perturbation. The primary approach used today, however, is based on inference from single cell-transcriptomic data. The transcriptome of a population of individual cells is measured at a given timepoint, and their transcriptional heterogeneity is quantitated using existing diversity indices. Transcriptional heterogeneity at a given point in time is assumed to be positively correlated with phenotypic plasticity of cells within the population. As the types of single cell phenotypic measures expands to include protein, chromatin, DNA methylation, *etc*., these inferences will undoubtedly become more sophisticated in deciphering the molecular underpinnings of this transcriptional diversity. Coupling these single cell measures with cell lineage barcoding or CRISPR based time recording approaches promise to allow phenotypic measurements in clonal lineages of cells over time, getting nearer to the ideal of unbiased live cell recording. Finally, there is clear evidence that the growth environment can influence the phenotype of plastic cancer cells. For example, mouse prostate organoid models of TSG deletion exhibit phenotypic plasticity but do not undergo neuroendocrine reprogramming *in vitro*. They do undergo neuroendocrine reprogramming when transplanted *in vivo*, demonstrating unique features of the *in vivo* tumor microenvironment contribute to NEPC reprogramming. Spatial transcriptomics at the single cell level will increasingly be used to assess the effects of the tumor growth environment on the phenotype assumed by plastic cancer cells, and vice versa.

Somatic genetic evolution clearly drives intratumoral heterogeneity that enables cancer progression and acquired therapeutic resistance. Given the varied, unpredictable, and highly selective pressures cancer cells face during disease progression, metastasis, and therapy, somatic genetic evolution alone may be insufficient to account for their remarkable adaptability. It is increasingly appreciated that epigenetic plasticity, a genetically encoded trait essential for normal development and homeostasis, can be aberrantly deployed by cancer cells to increase their adaptability. The next generation of precision and molecularly targeted therapies will need to address this plasticity to improve cancer patient outcomes and make further progress toward cures.

## Figures and Tables

**Table 1.** Phenotypic plasticity in homeostatic maintenance.

Tissue type	Plastic cell	Molecular drivers impacting plasticity	References
**Pancreas**	β islet cells α islet cells	Nanog*, Hes1 FoxO1, PRC2 loss	[26,29–32]
**Lungs**	Alveolar type II cells (AT2)	TGFβ*, Wnt7b Yap/Taz, BMP, p53*	[38–43]
**Intestines**	Crypt Base Columnar (CBC) Stem cells Paneth cells	Wnt/β catenin/Tcf4 Sox9, ASCL1*	[52–55]
**Liver**	Hepatocytes (HC) Biliary Endothelial cells (BEC)	Wnt/β catenin, Yap/Taz TGFβ*, Notch1*	[60–63]
**Skin**	Keratinocytes Adipocytes Hair Follicle (HF) Niche cells	Gata6, TGFβ*, Notch* Hedgehog, Wnt3a KLF5, Sox9 Slug*, Twist1*	[70,73,75,78,84–87]

* Directly associated with cancer phenotypic plasticity.

**Table 2.** Molecular pathways implicated in cancer phenotypic plasticity.

Cancer type	Lineage variant	Molecular drivers	References
**Prostate cancer**	Neuroendocrine Prostate Cancer (NEPC)	Rb1 loss, p53 loss, PTEN loss ASCL1, SOX2, FOXA2, MYC, ONECUT2, MUC1-C	[92–103]
	Double Negative Prostate Cancer (DNPC)	SOX2, FOXA2, ZNF263, KLF5	[104–106]
**Lung cancer**	Small Cell Lung Cancer (SCLC)	Rb1 loss, p53 loss, PTEN loss ASCL1, SOX2, MYC	[14,96,99,107,108]
	Squamous Cell Carcinoma (SCC)	LBK1 loss p63, AKT, MYC, JAK/STAT	[109–114]
	Epithelial-Mesenchymal Plasticity (EMP)	ASCL1, SNAIL, SLUG TWIST, ZEB1/2	[115,116]
**Melanoma**	Endothelial-like (Vasculogenic Mimicry)	β-catenin/TCF4 MYC, TWIST	[12,117,118]
**Breast cancer**	Basal/Mesenchymal Inflammatory Breast Cancer (IBC)	JAK/STAT, SOX10 ONECUT2, SNAIL, TGF-β	[13,17,119,120]
**Neuroblastoma**	Adrenergic-Mesenchymal Plasticity	SWI/SNF, MYCN, HAND2, PHOX2B, GATA3	[121]
**Pancreatic cancer**	Stem-like/EMP	BRD4, SOX2, SOX5, TWIST, NRF2	[122]
**Liver cancer**	Stem-like Hepatocellular Carcinoma (HCC)	SPINK1, E2F2	[123]
**Bladder cancer**	Urothelial-to-Squamous Trans differentiation	FOXA1 loss, GATA3 loss, PPARγ loss	[124]
**Leukemia**	B-cell Leukemia to T-cell / Myeloid Leukemia	Notch Signaling	[125,126]

## Abbreviations

AST: adeno-to-squamous transdifferentiation
AT1: alveolar type I
AT2: alveolar type II
AR: androgen receptor
ARSI: androgen receptor signaling inhibitors
ADT: androgen-deprivation therapy
BEC: biliary endothelial cells
CBC: crypt base columnar
DNPC: double negative prostate cancer
DNMT: DNA methyltransferase
DTP: drug tolerant persistor
EGFR TKI: EGFR tyrosine-kinase inhibitors
EGFR: epidermal growth factor receptor
EndMP: endothelial-mesenchymal plasticity
EMT: epithelial-mesenchymal transition
EMP: epithelial-mesenchymal plasticity
FSC: facultative stem cells
FGFR: fibroblast growth factor receptor
GEMM: genetically engineered mouse model
HF: hair follicle
HC: hepatocytes
HCC: hepatocellular carcinoma
HDAC: histone deacetylases
HMT: histone methyltransferases
IBC: inflammatory breast cancer
IPSCs: induced pluripotent stem cells
JAK: janus kinase
LIF: leukemia inhibitory factor
LUAD: lung adenocarcinoma
NEPC: neuroendocrine prostate cancer
PDAC: pancreatic ductal adenocarcinoma
PCa: prostate adenocarcinoma
STAT: signal transduction and transcription activation
SCLC: small cell lung cancer
TF: ranscription factor
TGF-β: transforming growth factor beta
TNBC: triple negative breast cancer
TSG: tumor suppressor genes
VM: vasculogenic mimicry
